# Supplementary material for: Superabundant microRNAs are transcribed from human rDNA spacer promoters insulated by CTCF
Source: Sci Adv. 2026 Mar 20;12(12):eaec1451. doi: 10.1126/sciadv.aec1451 (PMC13004009; doi:10.1126/sciadv.aec1451)
Supplement: Supplementary file 1 — Figs. S1 to S6 Legend for data S1 [file sciadv.aec1451_sm.pdf]

Science Advances
AAAS

Supplementary Materials for

Superabundant microRNAs are transcribed from human rDNA spacer promoters insulated by CTCF

Steven Henikoff and Jorja G. Henikoff

Corresponding author: Steven Henikoff, steveh@fredhutch.org

Sci. Adv. **12**, eaec1451 (2026)
DOI: 10.1126/sciadv.aec1451

The PDF file includes:

Figs. S1 to S6
Legend for data S1

Other Supplementary Material for this manuscript includes the following:

Data S1

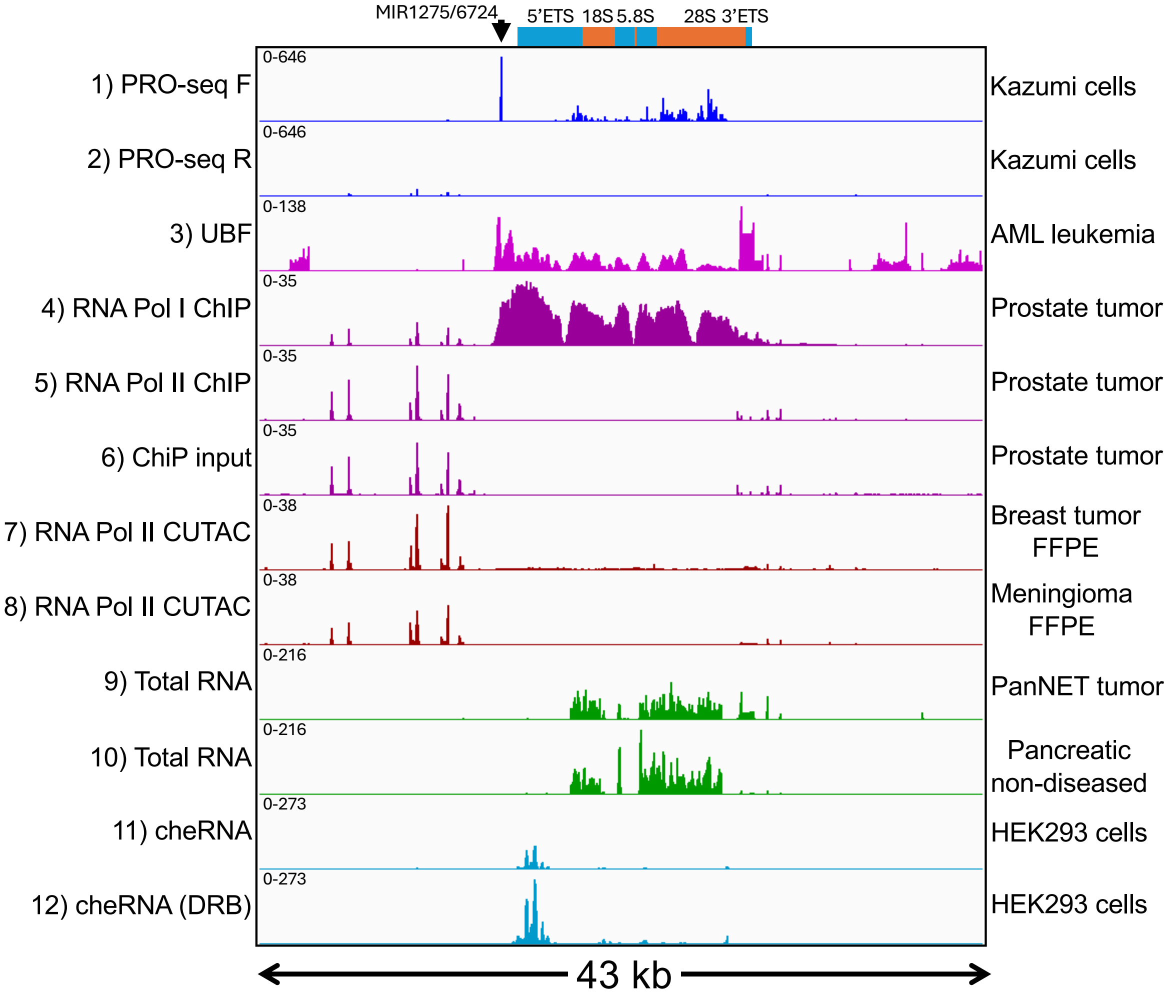

Fig. S1: Multi-modal profile alignments over the human rDNA repeat unit. PRO-seq forward stand signal is high over the transcribed region with a peak of maximum height just upstream. (**1-2**) PRO-seq data from Ref. (*25*) within a representative rDNA repeat unit (chr21:3,544,445-3,585,713); (**3**) UBF ChIP-seq data from GSM9095398; (**4-6**) Pol I and Pol II ChIP-seq data from GSM8267449; (**7-8**) RNA Pol II FFPE-CUTAC data from Ref. (*14*); (**9-10**) Total RNA data from Ref. (*11*); (**11-12**) chromatin-enriched RNA data from Ref. (*29*).

MIR10401
Human -29.36
CGUGUGGGAAGGCGUGGGUgcggaccccggcccg**ACCUCGCCGUCCCGCCCGCCG**
((.((((((.(((((.((((.(((...))))))).))...))).)))))).))...
Chimp -31.78 kcal/mol
CGUGUGGGGGAGGCGUGUGGGGUGCGGAGCCCGGCCCG**ACCUCGCCGUCCCGCCCG**
.....(((((.((((.(((((((.(((.((...)))))))))))))))))).))))).
Macaque -21.40 kcal/mol
GCGUUGGGAGGCACUGGGUguggaacccggcccg**ACCUCGCUGUCCCGACCCCG**
..(((((((((...(((((.(((........)))))))))).)))))))....

MIR663A
Human -50.00 kcal/mol
ccggcguccc**AGGCGGGGCGCCGCGGACCGC**ccucgugucuguggcggugggaucccgcggccguguuuccugguggcccggccgugc
((((.((((((((.((.((((((((((((((((.(((......))).)))))..)))))))).))).))..))))).)))))))......
Chimp -51.39 kcal/mol
ccggcgucccgggc**GGGGCGCCGCGGACCGCCCUC**gugucuguggcggugggaucccgcggccguguuuccugguggcccggccgugc
((((.((((((((.((.((((((((((((((((.(((......))).)))))..)))))))).))).))..))))).)))))))......
Macaque -47.82 kcal/mol
ccggcgucccgggc**GGGGCGCCGCGGACCGCCCUC**gugucuguggcggugggaucccgcggccguguuuccugguggcccggccgugc
((((.((((((((.((.((((((((((((((((.(((......))).)))))..)))))))).))).))..))))).)))))))......

MIR3648
Human -54.85 kcal/mol
uggugggggg**AGCCGCGGGGAUCGCCGAGGG**ccggucggccgccccgggugccgcgcggugccgccggcggcggugaggccccgcgcgugug
..(((.((((.((((((.((..((((..((((.((....))))))..)))))).))))))((((((...))))))....)))).))).....
Chimp -54.07 kcal/mol
uggugggggg**AGCCGCGGGGAGCGCCGAGGG**ccggucggccgcuccgggugccgcgcggggccgccuucccgcucggaggcugccggcggu
....((((.((..(((((.((.((.(((((((((....)))).)).))))).)).)))))..)).)))).((((.(((......))))))).
Macaque -46.50 kcal/mol
Ggcggtggggg**AGCCGCAGGGATCGCTGAAGG**cctggtcggccgctccaggtgccacgcggggccgccttcgtgctcggaggctgctggcgg
((((.(((((..((((..(((.((......)))))...))))..)))))..)))).(((..(((.(((((((....))))))).))).))).

MIR3687 (no hairpin in miRBase)
Human: **CCCGGACAGGCGTTCGTGCGACGT**
**********************+**
Chimp: **CCCGGACAGGCGTTCGTGCGATGT**
*****+****+*************
Macaque: **CCCGGGCAGGTGTTCGTGCGATGT**

MIR10396
Human -36.66 kcal/mol
GGCGGGGCUCGGAGCCGGGcuucggccg**GGCCCCGGGCCCUCGACCGGG**
(((((((((((.(((.(((....))).))).))))))))).)).))...
Chimp -41.45 kcal/mol
GGCGGGGCUCGGAGCCGGGcuucggccg**GGCUCCGGGCCCUCGACCGGA**
((((((((((((((((.(((....))).)))))))))))))).)).))...
Macaque -35.33 kcal/mol
GCGGGGCUCGGAGCCGGGCcucggccg**GGCCCCGGGCCUCGACCGGA**
.((((((((((.(((.(((....))).))).))))))))))......

Fig. S2: MicroRNA precursors in the 5'ETS region are present in other primates. RNAfold(*32*) was used to predict foldback structures for the five miRBase-annotated microRNAs in the 5'ETS region. Foldback structures are shown in Vienna format. expanded around the promoter and 5'ETS.

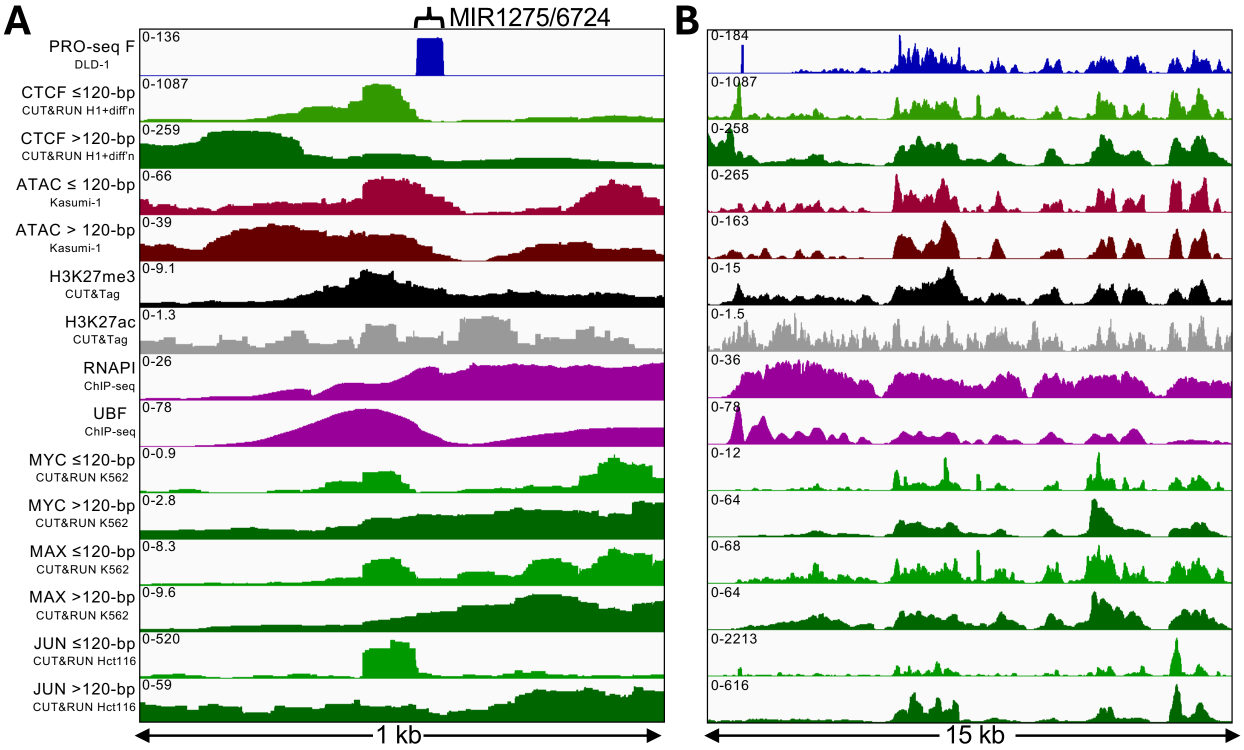

Fig. S3: Myc/Max and AP-1 Transcription Factors bind to the miR-1275/6724 promoter. Subnucleosomal CTCF fragments (≤120 bp, green) map immediately adjacent to the PRO-seq peak (blue) directly over the miR-1275/6724 DNA sequence and the major UBF peak, whereas nucleosomal CTCF peaks (>120 bp, dark green) map farther upstream, with little overlap. Merged reads are from human H1 embryonic cells and 3-4 days of differentiation. A similar pattern is seen for ATAC-seq reads (light and dark brown). In metastatic prostate cancer, the mark of Polycomb silencing, H3K27me3 (black), but not H3K27ac (grey) a mark of active chromatin, peaks over the miR-1275/6724-adjacent CTCF and UBF sites, which suggests that the shift of CTCF from direct binding to nucleosome binding upstream is accompanied by gain of an H3K27me3 nucleosome that silences the rDNA spacer promoters. K562 cell MYC, MAX and Hct116 JUN CUT&RUN fragments (light and dark brown) were profiled as controls and show co-occupancy with UBF and shift downstream. (**B**) Same as (A) expanded around the promoter and 5'ETS regions.

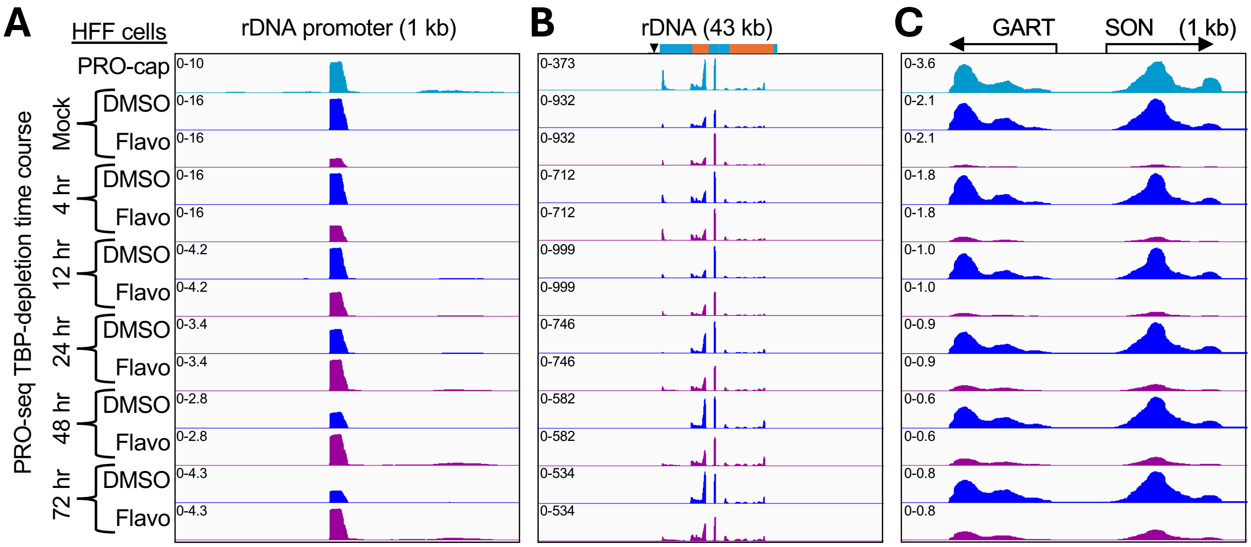

Fig. S4: Forward-strand PRO-cap and PRO-seq mini-chromosome profiles at the rDNA promoter (**A**), the full rDNA repeat (**B**), and the GART-SON control region (**C**), for DMSO- or Flavopiridol-treated cultures after PROTAC induction for the indicated times.(*64*) When TBP-depletion time points are autoscaled, dramatic PRO-seq signal increases after Flavopiridol treatment are seen for the miR-1275/6724, compared to modest decreases for the GART/SON bidirectional promoters and modest increases for rDNA transcription.

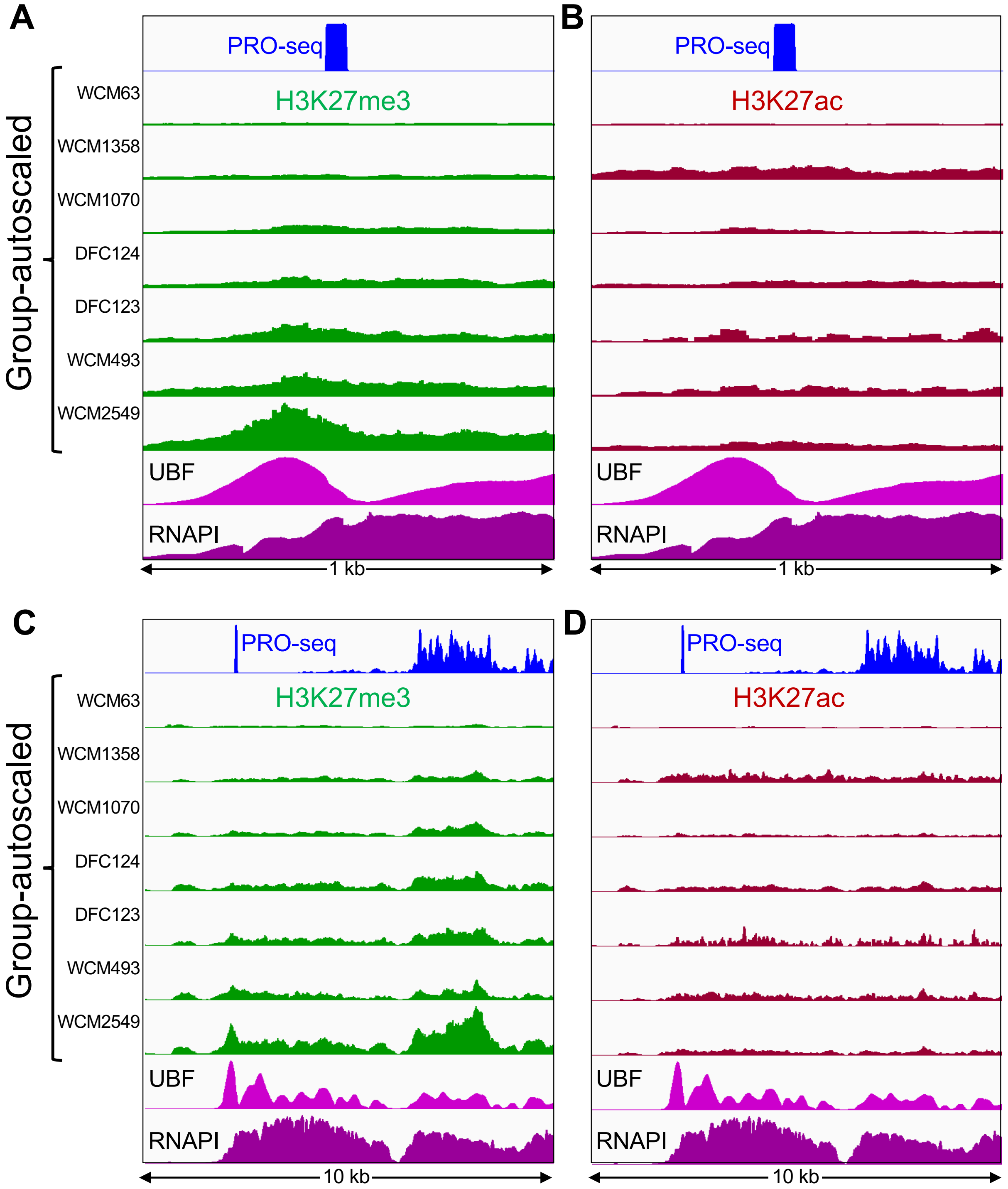

Fig. S5: H3K27me3-marked rDNA genes vary in abundance between prostate cancer metastases. High H3K27me3 (**A**) and low H3K27ac (**B**) over the Pol I-transcribed regions from prostate metastases are consistent with densely packed Pol Is on active copies and heterochromatic histone marks on silenced copies. CUT&Tag patient data from GSM8791938-GSM8791987 (Ref. (*55*)). Large variations in occupancy may reflect differences in both epigenetic and copy number differences between individuals.(*52*) (**C-D**) Same as (A-B) expanded around the promoter and 5'ETS regions.

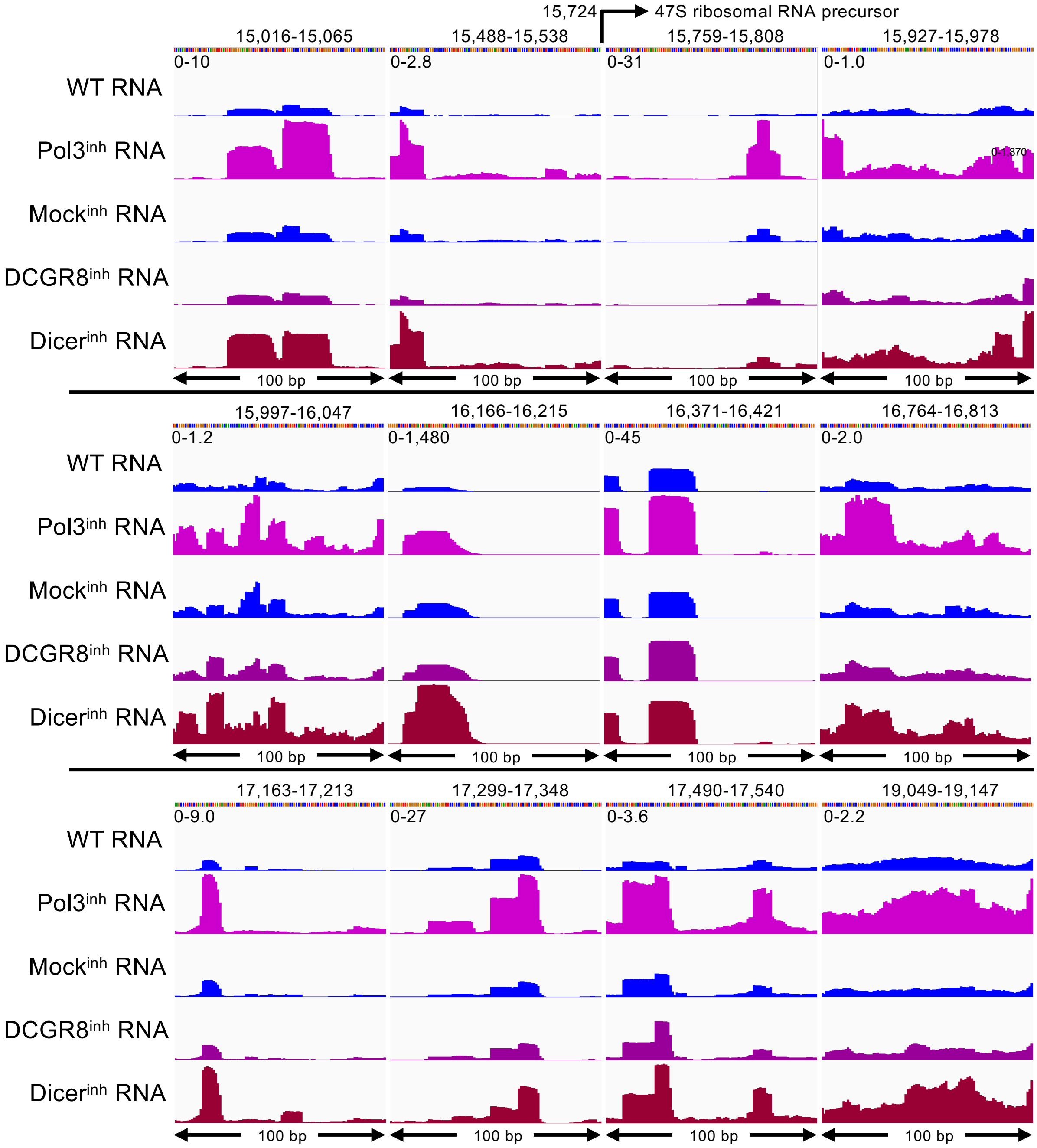

Fig. S6: Processed microRNAs from the rDNA promoter and 5'ETS use a non-canonical processing pathway. See the legend to Fig. 8, using data from RKO cells.(*57*) Group-autoscaled alignments were to the same 100-bp rDNA sub-regions used in the analysis shown in Fig. 6. Seven of the twelve microRNA precursors identified in NET-seq data show one or two characteristic fully processed microRNA peaks with signal counts ranging from 2.0 to 1,480, including miR-1275/6724, with 10 signal counts. Mir-4466 in region 16,371-16,421 is the only other annotated microRNA among those that show both NET-seq precursor signal and processed microRNA-seq signal. Inhibition of the DCGR8 Microprocessor subunit and the Dicer indicates that nearly all of these microRNAs do not use the canonical processing pathways.

Supplementary Data 1: **Reference sequence chm13_mini: Sequences from T2T-CHM13 version 2 (hs1) contain these "chromosomes":** GART-SON [chr21:31,912,598-31,959,322 (46725 bp)]**;** Hist_Chr1 [chr1:148,935,900-149,012,900 (77001 bp)]; Hist_Chr6 [chr6:25,878,304-26,154,469 (276166 bp)]; rDNA [hs1 chr21:3,141,168-3,185,955 (44788 bp)].
